
JOURNAL INFORMATION
==============================
NLM Title Abbreviation: CPT Pharmacometrics Syst Pharmacol
Journal ID: CPT Pharmacometrics Syst Pharmacol
NLM Journal ID: 101580011
Journal ID: PSP4

CPT: Pharmacometrics & Systems Pharmacology

EISSN: 2163-8306
Publisher: Wiley

ARTICLE INFORMATION
==============================
PMCID: PMC11474696
DOI: 10.1002/psp4.12986
Article ID: PSP412986
Article version: 1
Subjects: Issue Information, Issue Information

Issue Information

Electronic publication date: 2024 Mar 15
Publication date: 2024 Mar
Volume: 13
Issue: 3
First page: 331
Last page: 334
Copyright: © 2024 ASCPT
License: This is an open access article under the terms of the http://creativecommons.org/licenses/by/4.0/ License, which permits use, distribution and reproduction in any medium, provided the original work is properly cited.
License URL: https://creativecommons.org/licenses/by/4.0/

Figure count: 0
Table count: 0
Page count: 3

source-schema-version-number: 2.0
cover-date: March 2024
details-of-publishers-convertor: Converter:WILEY_ML3GV2_TO_JATSPMC version:6.4.9 mode:remove_FC converted:15.10.2024

==============================

An Official Journal of ASCPT and ISoP

### CPT: Pharmacometrics & Systems Pharmacology

Open Access

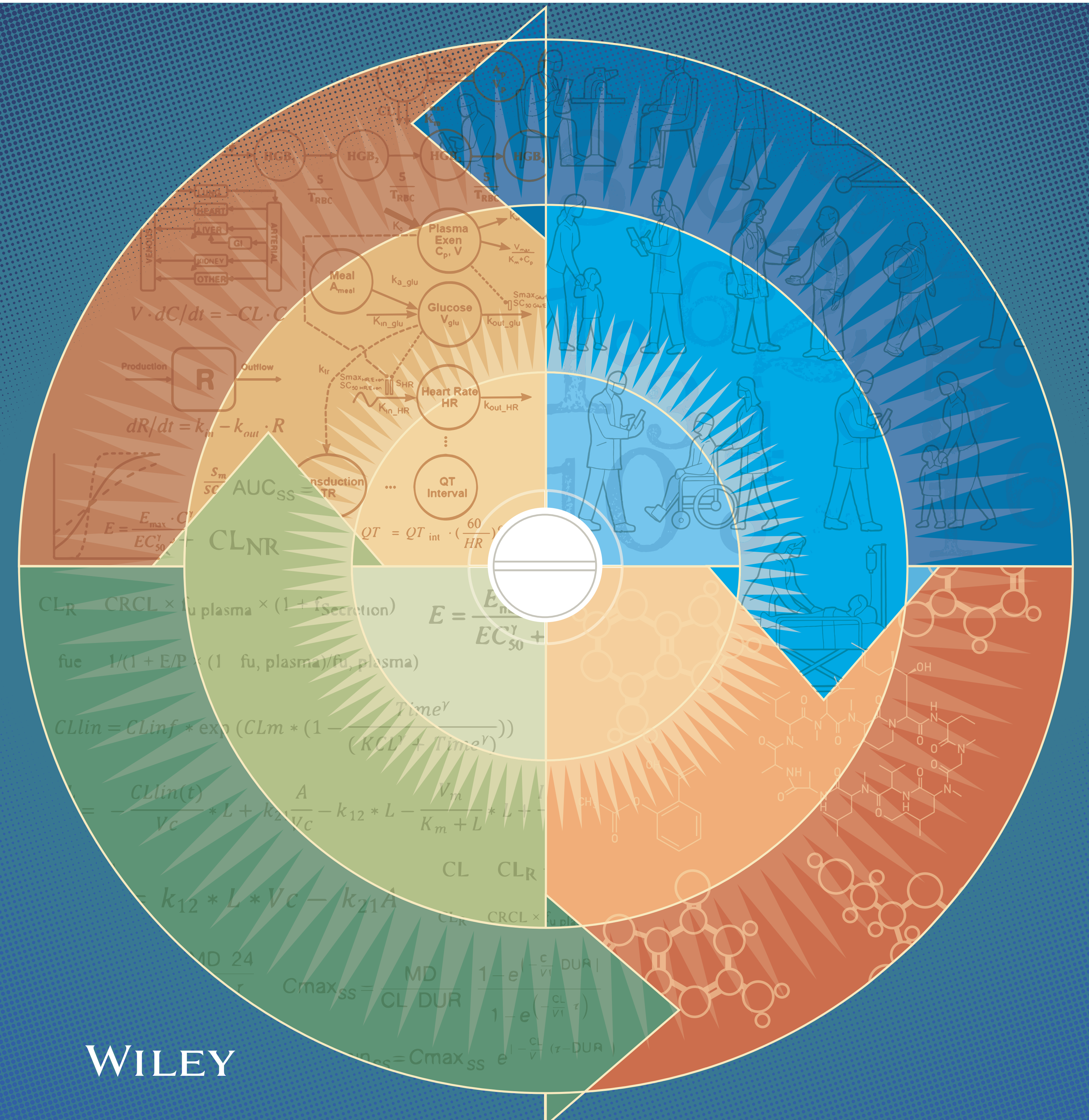

WILEY

### CPT: Pharmacometrics & Systems Pharmacology

WILEY

### Editor-in-Chief

France Mentré, MD, PhD, *School of Medicine of University Paris Diderot, Paris, France*

### Deputy Editor-in-Chief

Karen Rowland Yeo, PhD, *Certara UK Ltd., Sheffield, UK*

### Associate Editors

Paolo Denti, PhD, *University of Cape Town, Cape Town, South Africa*
Rajat Desikan, PhD, *GSK, Stevenage, UK*
Jonathan French, ScD, *Johnson & Johnson Innovative Medicine, Spring House, PA, USA*
Stefanie Hennig, MSc, PhD, *Certara Strategic Consulting, Brisbane, Australia*
Priya Jayachandran, PharmD, *Regeneron Pharmaceuticals, Inc., San Francisco, CA, USA*
Andrew Hooker, PhD, *Uppsala University, Uppsala, Sweden*
Sriram Krishnaswami, PhD, *Pfizer, Groton, CT, USA*
Ping Zhao, PhD, *Bill & Melinda Gates Foundation, Seattle, WA, USA*

### Editors-in-Training

Brian Cicali, PhD, *University of Florida, College of Pharmacy, Orlando, FL, USA*
Jane Knöchel, PhD, *AstraZeneca , Mölndal, Sweden*

### American Society for Clinical Pharmacology and Therapeutics

***Chief Executive Officer***
Abigail W. Gorman, *Alexandria, VA*

***Chief Operating Officer***
Elise S. Laffman-Johnson, *Alexandria, VA*

***Managing Editor***
Alethea B. Gerding, *Alexandria, VA*

***Deputy Managing Editor***
Alaina Webster, *Alexandria, VA*

***Senior Editorial Coordinator***
Aimee Greene, *Alexandria, VA*

### Editorial Board

Vivaswath S. Ayyar, PhD, *Janssen Research & Development, Spring House, PA*
Robert Bies, PharmD, PhD, *State University of New York at Buffalo, Buffalo, NY, USA*
Marc Birtwistle, PhD, *Clemson University Clemson, SC, USA*
Yuan Chen, PhD, *Genentech South San Francisco, CA, USA*
Marylore Chenel, PharmD, PhD, *Pharmetheus Uppsala, Sweden*
Emmanuel Chigutsa, PhD, *Eli Lilly and Company, Indianapolis, IN, USA*
Teresa Dalla Costa, PhD, *Federal University of Rio Grande do Sul - UFRGS Porto Alegre, Brazil*
Narendra M Dixit, PhD, *Indian Institute of Science, Bengaluru, India*
Anne-Gaelle Dosne, PharmD, PhD, *Janssen Research & Development, Beerse, Belgium*
Thomas Dumortier, Eng, MSc, *Novartis Pharma AG, Basel, Switzerland*
James M. Gallo, PharmD, PhD, *University at Buffalo, Buffalo, NY, USA*
Wei Gao, PhD, *EMD Serono, Billerica, MA, USA*
Jérémie Guedj, PhD, *INSERM, Paris, France*
Manish Gupta, PhD, FCP, *GenMab; Princeton, NJ, USA*
Bart Hendriks, PhD, *Novartis Institutes for BioMedical Research Cambridge, MA, USA*
Nick Holford, MBChB, *The University of Auckland Auckland, New Zealand*
Brett A. Howell, PhD, *DILIsym Services Division, Simulations Plus, Inc., Research Triangle Park, NC, USA*
Manuel Ibarra, PhD, *Universidad de la República, Montevideo, Uruguay*
Siv Jönsson, PhD, *Uppsala University Sweden, Uppsala, Sweden*
Kristin Karlsson, PhD, *Uppsala University Uppsala, Sweden*
Helen Kastrissios, PhD, *QuanTx Consulting, Brisbane, Australia*
Ron Keizer, PhD, *InsightRX San Francisco, CA, USA*
Steven E. Kern, PhD, *Global Health Labs, Bellevue, WA, USA*
Akash Khandelwal PhD, *UCB Biosciences GmbH, Monheim am Rhein, Germany*
Andrzej Kierzek, PhD, *Certara QSP; Sheffield, United Kingdom*
Qi Liu, PhD, *US Food and Drug Administration, Silver Spring, MD*
James Lu, PhD, *Genentech, Inc., South San Francisco, CA, USA*
Rajanikanth (Raj) Madabushi, PhD, *US Food and Drug Administration, Silver Spring, MD*
Efthymios Manolis, MSc, *European Medicines Agency London, United Kingdom*
Samer Mouksassi, PharmD, PhD, *Certara, Cairo, Egypt*
Jackson K. Mukonzo, PhD, *Makerere University, Kampala Uganda*
Joakim Nyberg, PhD, *Pharmetheus, Uppsala, Sweden*
Elsje Pienaar, PhD, *Weldon School of Biomedical Engineering, Purdue University, West Lafayette, IN, USA*
Daniel Scholes Rosenbloom, PhD, *Merck & Co., Rahway, NJ, USA*
Joe Standing, PhD, *University College London London, United Kingdom*
Natasha Strydom, PhD, *Amgen, Inc., San Francisco, CA, USA*
Yuichi Sugiyama, PhD, *Riken Innovation Center Sugiyama Laboratory Yokohama, Japan*
Elin Svensson, PhD, *Uppsala University, Uppsala, Sweden*
Joel Tarning, PhD, *Mahidol-Oxford Tropical Medicine Research Unit Bangkok, Thailand*
Nadia Terranova, PhD, *Merck KGaA, Lausanne, Switzerland*
Michael Tortorici, PharmD, PhD, *Aro Biotherapeutics, Philadelphia, PA, USA*
Coen van Hasselt, PhD, *Leiden University, Leiden, the Netherlands*
Paolo Vicini, PhD, MBA, *Confo Therapeutics, Ghent, Belgium*
Camille Vong, PhD, *Novartis AG Pharma, Basel, Switzerland*
Xinyuan (Susie) Zhang, PhD, *Daiichi Sankyo, Inc. Basking Ridge, NJ, USA*

### Overview

*CPT: Pharmacometrics & Systems Pharmacology* (PSP) is an official open access journal of the American Society for Clinical Pharmacology and Therapeutics (ASCPT) and International Society of Pharmacometrics (ISoP).

*CPT: Pharmacometrics & Systems Pharmacology* is a cross-disciplinary journal devoted to publishing advances in quantitative (e.g., modeling and simulation) methods as applied in pharmacology, physiology and therapeutics in humans. The journal welcomes original research articles, reviews and tutorials that bridge the following areas: pharmacometrics, modeling and simulation as applied to the design and evaluation of clinical trials, systems pharmacology modeling, particularly with a mechanistic link to human (patho)physiology, disease modeling, "population" or mixed-effects pharmacokinetics and pharmacodynamics (PKPD) modeling, modeling and simulation to support translational research, physiologically-based pharmacokinetics (PBPK), model-based meta-analyses of clinical trials, mechanism-based pharmacokinetic-pharmacodynamic modeling, computational pharmacology, bioinformatics, comparative efficacy, effectiveness and cost-effectiveness. Systems pharmacology may involve the application of systems biology approaches to study drug activities, targets and effects. The discipline is often defined with reference to engineering and pharmacological principles as the quantitative analysis of the dynamic interactions between drugs and a biologic system that aims to understand the behavior of the system as a whole. The common focus will be on quantitative methods that improve our understanding of pharmacology and therapeutics in humans.

**Abstracting and Indexing**
- Embase (Elsevier)
- SCOPUS (Elsevier)
- MEDLINE/PubMed indexing

**Society Information**
The American Society for Clinical Pharmacology and Therapeutics (ASCPT) was founded in 1900, and consists of over 2,200 professionals whose primary interest is to advance the science and practice of clinical pharmacology and translational medicine for the therapeutic benefit of patients and society. ASCPT is the largest scientific and professional organization serving the disciplines of Clinical Pharmacology and Translational Medicine.

**All articles published in this journal are freely available to read, download, and share. Articles are published under the terms of the Creative Commons Attribution Non-Commercial License which permits use, distribution, and reproduction in any medium, provided the original work is properly cited and is not used for commercial purposes.**

**Disclaimer**
The Publisher, the American Society for Clinical Pharmacology and Therapeutics, the International Society of Pharmacometrics, and Editors cannot be held responsible for any errors in or any consequences arising from the use of information contained in this journal. The views and opinions expressed do not necessarily reflect those of the Publisher, the American Society for Clinical Pharmacology and Therapeutics, the International Society of Pharmacometrics or Editors, neither does the publication of advertisements constitute any endorsement by the Publisher, the American Society for Clinical Pharmacology and Therapeutics, the International Society of Pharmacometrics, Editors, or Authors of the products advertised.

**Publisher**
*CPT: Pharmacometrics & Systems Pharmacology* is published by Wiley Periodicals, LLC, 101 Station Landing, Suite 300, Medford, MA 02155; Telephone: 781-388-8200; Fax: 781-388-8210.

CONTENTS

CPT: Pharmacometrics & Systems Pharmacology

### PERSPECTIVE

### PERSPECTIVE

**335** The interaction of core modules as a basis for elucidating network behavior determining Parkinson's disease pathogenesis
**Govind Menon, Suruchi Bakshi and J. Krishnan**

### TUTORIAL

### TUTORIAL

**341** gPKPDviz: A flexible R shiny tool for pharmacokinetic/pharmacodynamic simulations using mrgsolve
**Tong Lu, Victor Poon, Logan Brooks, Erick Velasquez, Eric Anderson, Kyle Baron, Jin Y. Jin and Matts Kågedal**

### RESEARCH

### ARTICLES

**359** *In silico* trial for the assessment of givinostat dose adjustment rules based on the management of key hematological parameters in polycythemia vera patients
**Elena M. Tosca, Alessandro De Carlo, Roberta Bartolucci, Francesco Fiorentini, Silvia Di Tollo, Maurizio Caserini, Maurizio Rocchetti, Paolo Bettica and Paolo Magni**

**374** Power to identify exposure-response relationships in phase IIa pulmonary tuberculosis trials with multi-dimensional bacterial load modeling
**Simon E. Koele, Thomas P. C. Dorlo, Caryn M. Upton, Rob E. Aarnoutse and Elin M. Svensson**

**386** Supplementing clinical lactation studies with PBPK modeling to inform drug therapy in lactating mothers: Prediction of primaquine exposure as a case example
**Xian Pan, Khaled Abduljalil, Lisa M. Almond, Amita Pansari and Karen Rowland Yeo**

**396** Evaluation of the potential impact on pharmacokinetics of various cytochrome P450 substrates of increasing IL-6 levels following administration of the T-cell bispecific engager glofitamab
**Nassim Djebli, Neil Parrott, Felix Jaminion, Amaury O'Jeanson, Elena Guerini and David Carlile**

**410** Clofazimine pharmacokinetics in HIV-infected adults with diarrhea: Implications of diarrheal disease on absorption of orally administered therapeutics
**Cindy X. Zhang, Thomas M. Conrad, David Hermann, Melita A. Gordon, Eric Houpt, Pui-Ying Iroh Tam, Khuzwayo C. Jere, Wilfred Nedi, Darwin J. Operario, Jacob Phulusa, Gerald V. Quinnan Jr, Leigh A. Sawyer, Lynn K. Barrett, Herbert Thole, Neema Toto, Wesley C. Van Voorhis and Samuel L. M. Arnold**

**424** Virtual twins for model-informed precision dosing of clozapine in patients with treatment-resistant schizophrenia
**Sam Mostafa, Reza Rafizadeh, Thomas M. Polasek, Chad A. Bousman, Amin Rostami-Hodjegan, Robert Stowe, Prescilla Carrion, Leslie J. Sheffield and Carl M. J. Kirkpatrick**

**437** Tumor growth inhibition-overall survival modeling in non-small cell lung cancer: A case study from GEMSTONE-302
**Yucheng Sheng, Shu-wen Teng, Jingru Wang, Hao Wang and Archie N. Tse**

**449** Microglial roles in Alzheimer's disease: An agent-based model to elucidate microglial spatiotemporal response to beta-amyloid
**Catherine Weathered, Sophia Bardehle, Choya Yoon, Niyanta Kumar, Cheryl E. G. Leyns, Matthew E. Kennedy, Peter Bloomingdale and Elsje Pienaar**

**464** Development and application of neonatal physiology-based pharmacokinetic models of amikacin and fosfomycin to assess pharmacodynamic target attainment
**Christopher A. Darlow, Neil Parrott, Richard W. Peck and William Hope**

**476** Nivolumab and ipilimumab population pharmacokinetics in support of pediatric dose recommendations—Going beyond the body-size effect
**Zheyi Hu, Sihang Liu, Yue Zhao, Shengnan Du, Lora Hamuro, Jun Shen, Amit Roy and Li Zhu**

**494** Population pharmacokinetics of the GIP/GLP receptor agonist tirzepatide
**Karen Schneck and Shweta Urva**
